# A parallel CUDA implementation of the Gauss–Legendre–spherical-*t* method for electrostatic interactions

**DOI:** 10.1063/5.0264935

**Published:** 2025-06-09

**Authors:** James E. Gonzales, Wonmuk Hwang, Bernard R. Brooks

**Affiliations:** 1 Department of Biomedical Engineering, Texas A&M University, College Station, Texas 77843, USA; 2 Laboratory of Computational Biology, National Heart, Lung, and Blood Institute, National Institutes of Health, Bethesda, Maryland 20892, USA; 3 Department of Materials Science and Engineering, Texas A&M University, College Station, Texas 77843, USA; 4 Department of Physics and Astronomy, Texas A&M University, College Station, Texas 77843, USA; 5 Center for AI and Natural Sciences, Korea Institute for Advanced Study, Seoul 02455, Republic of Korea

## Abstract

Computing electrostatic interactions remains the bottleneck of molecular dynamics (MD) simulations despite more than a century of effort in developing methods to accelerate the calculation. Previously, we have developed the spherical grids and treecode and Gauss–Legendre–spherical-*t* (GLST) algorithms for electrostatic interactions. Here, we explain the computational details and discuss the performance of GLST. The GLST algorithm achieves O(N) scaling and should be less demanding in parallel communication compared with the widely used particle mesh Ewald method and likely comparable to the communication costs of the fast multipole method. We find that GLST is suitable for rapid calculation of long-range electrostatic interactions in MD simulations as it has highly tunable accuracy and should scale well on massively parallel computing architectures. The GLST software presented here is available as a standalone library on GitHub.

## I. INTRODUCTION

Molecular dynamics (MD) simulations are a powerful tool that enables the atomic-level study of biological systems.^1^ Over many years of development and with significant improvements in computer hardware, MD simulations have been used to study a variety of systems of increasing size and complexity.^1^ Despite considerable advances, computing electrostatic interactions has consistently remained the rate-limiting step in the efficiency of MD engines. Electrostatic interactions are costly to compute as a result of the slow 1/*r* decay and poor $O\left(N^{2}\right)$ scaling of the Coulomb potential. Algorithms such as the fast multipole method (FMM)^2–4^ and particle mesh Ewald (PME) method^5–8^ accelerate the calculation by splitting the Coulomb potential into short- and long-range components in Ewald-based methods or near- and far-field terms in FMM.^9^

Both FMM and PME provide significant performance improvements, as the algorithms achieve O(N) and O(N⁡log⁡N) scaling, respectively.^3,4,7,8^ However, in spite of the performance gains over the traditional Coulomb potential, both FMM and PME have limitations. While PME is the standard method used in most MD simulations, the algorithm becomes extremely inefficient when implemented on large, multi-node machines as a consequence of the large amount of communication required by the three-dimensional (3D) fast Fourier transform.^10–12^ While FMM is able to achieve linear scaling, it comes at the cost of a sophisticated computational prefactor calculation, which prevents it from being used over PME for most systems.^3,4^

In a previous paper, we have developed the Spherical Grids and Treecode (SGT) algorithm that combines the Gauss–Hermite quadrature and spherical-t design points (herein referred to as a cubature) to evaluate the long-range component of the Ewald factorization of the Coulomb potential.^13^ Based on this, we further developed the Gauss–Legendre–spherical-*t* method (GLST) as a method that can be more practically implemented.^14^ Here, we give a brief overview of the GLST algorithm, outline the computational details of our implementation, and present the performance and accuracy of the method.

## II. THEORY

In the GLST algorithm,^14^ the Coulomb potential is split into short-range (erfc) and long-range (erf) components,

$$\frac{q_{a}q_{b}}{\left|{\vec{r}}_{ab}\right|}=\frac{q_{a}q_{b}\text{erfc}\left(\alpha \left|{\vec{r}}_{ab}\right|\right)}{\left|{\vec{r}}_{ab}\right|}+\frac{q_{a}q_{b}\text{erf}\left(\alpha \left|{\vec{r}}_{ab}\right|\right)}{\left|{\vec{r}}_{ab}\right|},$$ (1)

where $\text{erf}\left(\cdot \right)$ and $\text{erfc}\left(\cdot \right)$ are the error function and complementary error function, respectively, *α* is the Ewald convergence parameter,^9^ and $\left|{\vec{r}}_{ab}\right|$ is the distance between two interacting atoms *a* and *b*. The short-range term is handled through direct, pairwise interactions, and the long-range term is handled through GLST. The simulation box is divided into cells, and the interaction energy between atom *a* in a cell with atoms *b* in a non-neighboring cell (C) is

$$E_{a}^{C}=q_{a}\underset{b\in C}{\sum }q_{b}\frac{\text{erf}\left(\alpha \left|{\vec{r}}_{ab}\right|\right)}{\left|{\vec{r}}_{ab}\right|}.$$ (2)

The GLST cubature expresses the long-range potential in terms of the roots (*s*_*n*_) and weights (*w*_*n*_) of the Gauss–Legendre quadrature, a finite integration cutoff (*ζ*), and spherical t-design points $\left({\hat{t}}_{m}\right)$. The finite integration cutoff *ζ* truncates the integral expression of the long-range interaction term to a finite range to use the Gauss–Legendre quadrature and to focus on the interval that contributes meaningfully to the integral provided a given target accuracy,^14^

$$\begin{array}{ll} E_{a}^{C} & =q_{a}\overset{N_{l}}{\underset{n}{\sum }}\frac{2\alpha \zeta }{\pi M_{t}}w_{n}\exp\left(-\zeta^{2}s_{n}^{2}\right)\overset{M_{t}}{\underset{m}{\sum }}\exp\left(2\alpha i\zeta s_{n}{\hat{t}}_{m}\cdot {\vec{r}}_{a}\right)\\ & \;\times \underset{b\in C}{\sum }q_{b}\exp\left(-2\alpha i\zeta s_{n}{\hat{t}}_{m}\cdot {\vec{r}}_{b}\right)+\varepsilon , \end{array}$$ (3)

where *N*_*l*_ is the number of positive roots in the Gauss–Legendre quadrature, *M*_*t*_ is the number of points in the upper hemisphere of the spherical t-design grid, and *ɛ* is the error. For computational efficiency, we express the complex exponentials as the real and imaginary trigonometric terms, and for a given atom, we define a basis function at a specific cubature node (*α*, *n*, *m*) as follows:

$$\begin{array}{c} C_{mn}^{a}=q_{a}\cos\left(2\alpha \zeta s_{n}{\hat{t}}_{m}\cdot {\vec{r}}_{a}\right),\\ S_{mn}^{a}=iq_{a}\sin\left(2\alpha \zeta s_{n}{\hat{t}}_{m}\cdot {\vec{r}}_{a}\right). \end{array}$$ (4)

The basis functions of all the atoms within a cell can then be summed to compute the cosine and sine structure factors for the cell at a specific cubature node,

$$\begin{array}{c} C_{mn}^{C}=\underset{a\in C}{\sum }C_{mn}^{a},\\ S_{mn}^{C}=\underset{a\in C}{\sum }S_{mn}^{a}. \end{array}$$ (5)

The interaction energy ($E_{a}^{C}$) between an atom and all atoms in a distant cell can then be calculated by summing over all of the atom’s basis functions multiplied by the cell’s structure factor,

$$E_{a}^{C}=\frac{2\alpha \zeta q_{a}}{\pi M_{t}}\overset{N_{l}}{\underset{n}{\sum }}w_{n}\exp\left(-\zeta^{2}s_{n}^{2}\right)\overset{M_{t}}{\underset{m}{\sum }}C_{mn}^{a}C_{mn}^{C}-S_{mn}^{a}S_{mn}^{C}+\varepsilon .$$ (6)

## III. METHODS

In the following, we explain some of the technical details regarding the CUDA implementation of the GLST algorithm. The steps of the algorithm are divided into two groups: geometry-independent and geometry-dependent tasks. Geometry-independent tasks can be performed once during the initial setup and will only need to be updated if the box dimension changes. The geometry-dependent tasks need to be performed during each step of a MD simulation. The GLST algorithm requires a minimum input of (1) the number of atoms in the system, (2) a target error threshold value, (3) dimensions of the simulation unit cell, and (4) a pairwise interaction cutoff distance.

### A. Geometry-independent initialization

#### 1. Determining *α* groups

One of the main differences between the GLST^14^ and SGT^13^ algorithms is using multiple cubatures. In SGT, a single cubature is used and rescaled by powers of two, depending on the interaction distance. In GLST, unique cubatures are used to cover specific distance ranges, as opposed to simply scaling a single cubature. This is done to minimize the total number of cubature nodes that are needed to evaluate the long-range energies and forces, as the number of cubature nodes required for a given level of accuracy quickly decays with increasing distance.^14^ Each cubature can guarantee accuracy up to a given input tolerance for a specific range of distances and the corresponding Ewald convergence parameter (*α*). To this end, based on the size of the unit cell and the desired cutoff value, we can determine the number of *α* groups (or cubatures) and the corresponding distance ranges and *α* parameters for each group.

**FIG. 1.**
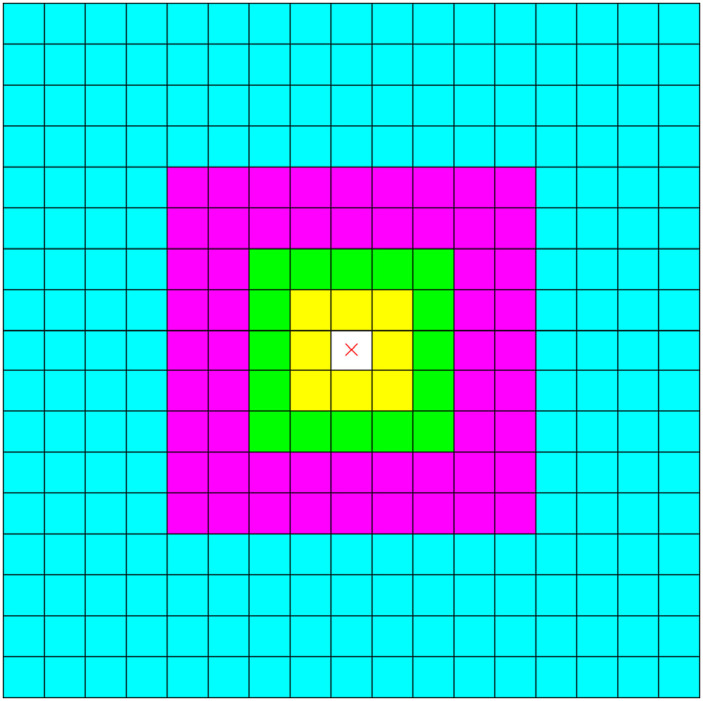
Two-dimensional depiction of *α* groups. The target cell is shown in white with a red “x.” The nearest neighbor cells (shown in yellow) interact with the target cell using direct, pairwise interactions. The green, magenta, and cyan cells interact with the target cell according to Eq. (2) and make up the first, second, and third *α* groups, respectively. The *α* groups comprise shells with increasing thickness. The shells formed by the green, magenta, and cyan cells are of thickness 1, 2, and 4, respectively.

Using the dimensions of the system and pairwise interaction cutoff, which are provided as the input, the system is divided into cubic cells, each with a side length of the pairwise cutoff. When evaluating energies and forces, each cell interacts with its 26 nearest neighbors using pairwise Coulomb interactions. Outside of this so-called “shell” of short-range neighbors, the target cell interacts with all other cells according to Eq. (2). To determine the number of *α* groups, we must consider shells of cells starting from two cells away, up to the total number of cells along one axis minus two. These shells comprise cells that surround the previous shell. A 2D depiction of the *α* grouping can be seen in Fig. 1. The first *α* group contains the shell of cells outside of the short-range neighbors (the green region in Fig. 1). After this initial group, the width of the shell for each *α* group is approximately doubled until all cells are accounted for. The minimum and maximum distances of each group are used to determine the *α* value, integration cutoff *ζ*, and the associated cubature to ensure long-range interactions involving cells in this shell are accurate within *ɛ*.

#### 2. Cell neighbor list

Whether two cells interact via short- or long-range potentials and which cubature to use for the long-range interactions are dependent on the size of each cell and the distance between the two cells. Unless cells change sizes, the potential or cubature that is used to interact two cells remains the same, thus allowing for a single cell neighbor list to be computed before dynamics.

The cell neighbor list is constructed in a similar manner as the *α* groups. For each cell in the system, shells of increasing thickness are assigned as either short-range neighbors or long-range neighbors for a given *α* group, as demonstrated in Fig. 1. The shell of neighboring cells immediately surrounding the current cell is assigned as short-range neighbors, and then, the one-cell-thick shell of cells around the short-range neighbors is assigned as long-range neighbors using the first cubature. Similar to the *α* groups, this process is repeated while increasing the shell thickness until all other cells in the unit cell are assigned.

#### 3. Cubature initialization

Initializing the cubatures requires the target error threshold that is provided as user input, the number of *α* groups, and the corresponding *α*, distances ranges, and integration cutoff values that are associated with a given *α* group. Provided these inputs, a suitable Gauss–Legendre quadrature and spherical t-design grid are chosen to achieve the target error [*ɛ* in Eqs. (3) and (6)]. As an optimization, the Gauss–Legendre roots and weights are combined with the spherical t-design points, resulting in what we refer to as the cubature nodes and weights. This is done to transform the two sums over *N*_*l*_ and *M* elements in Eq. (3) into a single sum over *N*_*l*_*M* elements, avoiding nested loops and accessing multiple arrays. In addition, constants such as *α* and *z* are also pre-incorporated to minimize the number of instructions done later when computing energies and forces.

#### 4. Memory allocation

In addition to the previously described initializations, all of the remaining memory allocation is performed. The CUB library from NVIDIA is used to perform sorting (see Sec. III B 1). This library requires some additional memory to be allocated, so calls to the sorting functions are done to determine the largest needed size of the work buffer.

### B. Geometry-dependent calculations

#### 1. Assign atoms to cells

The goal of GLST is to substitute computing long-range pairwise interactions with atom-to-cell interactions. To this end, atoms must be assigned to the correct cells based on their positions. We accomplish this by sorting the atom data by the associated cell indices. First, a cell index is calculated for each atom based on its current position. This index determines which cell will include the atom’s basis functions in the cell’s structure factor. During this step, the number of atoms in each cell is also calculated. Properties of each atom including the three Cartesian coordinates and forces, charge, and energy are stored in the respective one-dimensional arrays partitioned by the corresponding cells.

Having optimal memory access patterns is critical to the performance of memory-bound, GPU-based programs. In the GLST algorithm, multiple passes through arrays containing the atomic data are needed for computing the structure factors for cells. Atoms that are grouped together in a cell are likely somewhat randomly distributed throughout memory, so to avoid our implementation being memory-bound, we sort the coordinates and charges to allow coalesced memory access to the atoms that are assigned to a specific cell. To avoid performing multiple sorts, the atom coordinates, charges, and indices are packed into a structure and are then sorted using cell indices as keys. The sorting is performed by the NVIDIA CUB library, where we use the SortPairs routine of DeviceRadixSort. After the atom data have been sorted by the cell index, they are unpacked and stored in separate arrays. This sorting ensures optimal memory access when computing the structure factors for the cells, enabling us to fully utilize available hardware compute resources. Furthermore, the sorting of the atom data by their respective cells is an extremely fast operation taking about 3 ms for our 1.5 × 10^6^ atom system, so the sorting cost is amortized when computing the structure factors.

#### 2. Compute cell structure factors

When the interaction energy or forces are computed between two cells, the structure factor for the cells is used. This provides a substantial speed up over pairwise interactions as all atoms within a cell can interact with another atom in a different cell at once.^13^ Each cell in the system can compute its structure factor simultaneously. To best exploit this, we take advantage of dynamic parallelism, where a CUDA kernel launches many kernels from the device. A single driver kernel launched from the host launches separate kernels for each cell to compute its own structure factor. This is done to minimize the initial overhead of kernel launches upon startup. While kernel launch overhead is usually masked after many kernels have been queued, in our testing, we found that the overhead can be masked further by using dynamic parallelism. Furthermore, since atoms were sorted by the cell index in the previous step, memory access to both the cell’s atom data and the cubature nodes is coalesced.

To best utilize the available compute resources on the GPU, every cubature node for a given cell’s structure factor is assigned to a single warp. Each warp then iterates through the atoms that have been assigned to a given cell, computing a basis function for each atom. Each thread computes a partial contribution to the cell’s total structure factor by summing the basis functions that it calculates. After all atoms on the cell have been iterated, each warp performs a reduction of the partial structure factors to compute the total structure factor for the cell. The root lane thread in each warp then writes the final structure factor to the global memory.

#### 3. Sum remote structure factors

Each cubature has tens of thousands of nodes, so in an effort to prevent the memory requirements from becoming prohibitively large for big systems, the basis functions of each atom are recomputed as needed. This requires a sine and cosine evaluation for each cubature node, so to minimize the number of times that the basis functions are computed to two, we iterate through all of the long-range neighbors of each cell and sum the structure factors of the remote cells. This summed structure factor is then used to calculate the long-range energy so the basis functions for each atom can be reused.

#### 4. Compute long-range energies and forces

Computing the long-range energies and forces follows the same approach used for the cell structure factor calculation. Dynamic parallelism is used again, so a single kernel launched from the host launches a kernel for each cell. To compute the long-range energy and force, an atom is assigned to a warp. The warps then iterate through all of the cubature nodes and compute the basis function and the partial contribution to the total energy and force for a given atom. After all cubature nodes have been iterated, the warp performs another reduction to compute the total interaction energy and force for an atom within a given cell.

## IV. RESULTS

### A. Benchmarking details

We analyzed how the size of the system, target error threshold, and direct space cutoff impact the performance and accuracy of GLST. Our systems included neutral salt water boxes of varying sizes containing Na^+^ and Cl^−^ ions at varying concentrations. Details of each system are outlined in Table I. We refer to the runtime of the algorithm as the amount of time taken to compute the energy and forces of a system one time and do not include the time taken during initialization and memory allocation. The runtime was taken as the average over 50 energy and force calculations. To evaluate the accuracy of our method, we compared the energies and forces with a direct pairwise interaction using the Coulomb potential. The target error threshold provided as input is only used to determine the cubature needed to achieve the desired accuracy. The root mean square error (RMSE) that is calculated is the outcome of comparing the energy and forces computed by GLST to those computed by the Coulomb potential. While the GLST implementation uses double-precision arithmetic to maintain accuracy, we used single-precision trigonometric functions when computing the basis functions. Errors for both single- and double-precision trigonometric can be found in the supplementary material. All benchmarking was performed using an NVIDIA A100 GPU with 108 streaming multiprocessors.

**TABLE I.** Outline of the various neutral salt water boxes used for benchmarking. The number of water molecules, sodium and chloride ion pairs, total number of atoms, and box dimensions are outlined for the systems that were used for benchmarking.

No. of H_2_O	No. of NaCl	No. of atoms	Box dims. (Å)
12	1	38	8 × 8 × 8
115	2	349	16 × 16 × 16
979	11	2959	32 × 32 × 32
8172	84	24 684	64 × 64 × 64
65 275	667	197 159	128 × 128 × 128
101 212	1033	305 702	148 × 148 × 148
159 746	1631	482 500	172 × 172 × 172
222 303	2269	671 447	192 × 192 × 192
316 376	3229	955 586	216 × 216 × 216
411 904	4204	1 244 120	236 × 236 × 236
526 044	5368	1 588 868	256 × 256 × 256

### B. System size dependence

**FIG. 2.**
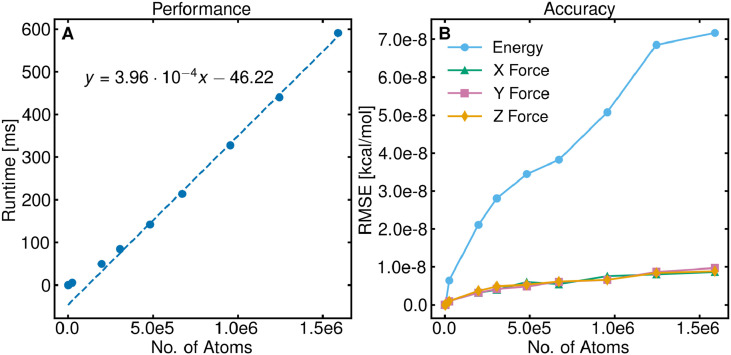
Dependence of the performance of GLST on system size. (a) Runtime of one energy and force calculation with a target accuracy threshold of 10^−4^. The solid circles denote the measured values, and the dashed line denotes a linear fit, revealing O(N) scaling. (b) RMSE of the energy and each component of the force with a target error threshold of 10^−8^.

We used cubic water boxes ranging in size from 38 to 1 588 868 atoms (see Table I). To determine the runtime [Fig. 2(a)], a target error threshold of 10^−4^ was used and multiple direct space cutoff distances were tested to find the optimal cutoff (see Sec. IV D). For demonstrating control over the accuracy [Fig. 2(b)], we used a stricter target error threshold value of 10^−8^ and a constant direct space cutoff of 20 Å (cell dimension) for all systems to ensure that the accuracy would only depend on the system size.

As seen in Fig. 2(a), GLST achieves $O\left(N\right)$ scaling as a result of its cubature and domain decomposition scheme. The RMSE of the energy and three components of the force all meet the target error threshold of 10^−8^ [Fig. 2(b)]. The slight increase in RMSE with system size is a consequence of the cubature being used, as more energies and forces are being approximated by several different cubatures, as described in Sec. III B, each with different errors on the order of the target error threshold value. Despite the errors from multiple cubatures, the RMSE is still on the order of 10^−8^ kcal/mol even for the largest system used containing 1.5 × 10^6^ atoms. The overall higher RMSE in the energy compared with force is because the latter decays faster with distance. When comparing normalized energy and force errors, they are found to be comparable (Tables S1 and S2).

We also computed histograms of the per-atom error for representative systems to understand our distribution of errors (Figs. S1 and S2). While maximum errors for the energies and forces slightly exceed the target error threshold, they are clustered around 0, resulting in a desirable RMSE.

### C. Target error threshold dependence

**FIG. 3.**
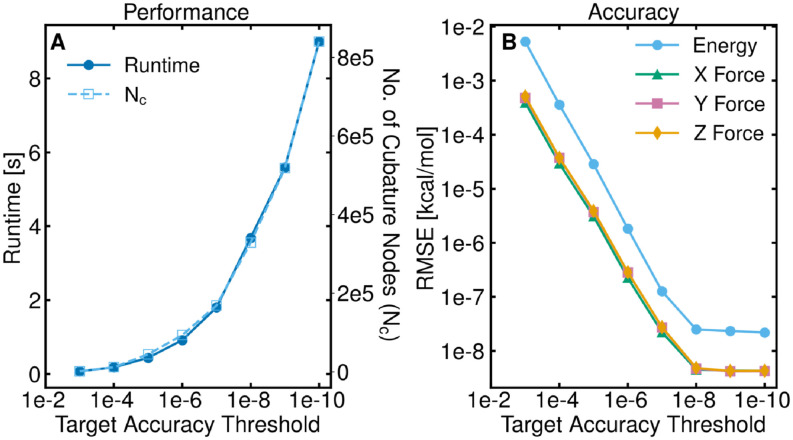
GLST dependence on the target error threshold. (a) Runtime (solid circles) and the number of cubature nodes used (open squares) for a given target error threshold. (b) RMSE in the energy and each component of the force vs the target error threshold.

Here, we analyze how the target error threshold impacts GLST. For these results, a salt water box containing 65 275 waters and 667 sodium and chlorine pairs (197 159 total atoms) was used with a direct space cutoff of 18 Å. From Fig. 3(a), it is clear that a higher target accuracy (smaller target error threshold) negatively impacts the performance, which is a direct consequence of more cubature nodes being needed to achieve the higher levels of accuracy. The cubature nodes have such a large impact on the runtime because for each atom, the basis functions must be computed at all of the cubature nodes. While the current runtime for very low errors $\left(\leq 10^{-8}\right)$ is prohibitively large to be used for MD simulations, the runtime for more realistic errors $\left(∼10^{-4}\right)$ is reasonable.

As the target error threshold decreases, the RMSE decreases until roundoff error becomes the limiting factor [Fig. 3(b)]. We also generated histograms of the per-atom error in the energies and forces (Figs. S3 and S4) and find that the distribution of errors remains Gaussian for different target error thresholds, while the overall error and spread decreases, further demonstrating the control of the error in the GLST algorithm. Similar to the size-dependent errors, the normalized errors remain comparable until a target accuracy threshold of 10^−8^ (Tables S3 and S4). After this point, the single precision trigonometric functions used (cf. Sec. IV E) do not have enough precision to achieve higher accuracies.

### D. Cutoff dependence

**FIG. 4.**
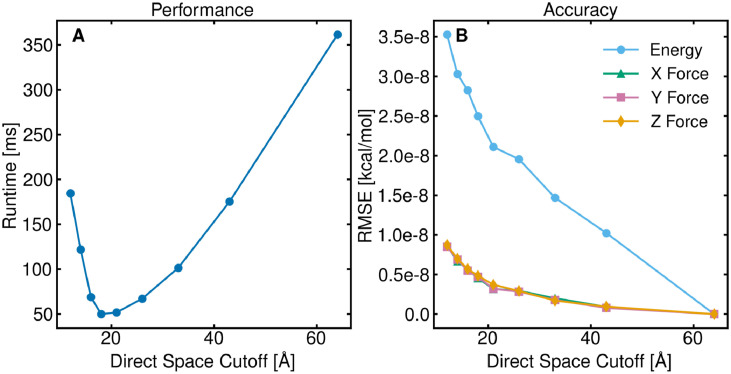
Dependence of the GLST performance on the pairwise interaction cutoff. A salt water box containing 197 159 atoms was used. (a) Runtime for various cutoff distances with a target error threshold of 10^−4^. (b) Root mean square error of the algorithm for various cutoff distances with a target error threshold of 10^−8^.

The last property we analyzed was the dependence on the direct space cutoff distance. We used the same 197 159-atom salt water box used for studying the dependence of the target error threshold above. Similar to Secs. IV B and IV C, target error thresholds of 10^−4^ and 10^−8^ were used for determining the runtime and accuracy, respectively. Similar to both FMM and PME, there is an optimal direct space cutoff distance for GLST [Fig. 4(a)].^3,8^ The optimal cutoff arises from the balancing of the short-range and long-range components. Above the optimal cutoff distance, calculation of short-range pairwise interactions dominates. On the other hand, below the optimal cutoff distance, the long-range term dominates as a result of a larger number of cubature nodes needed to achieve the target error threshold for smaller distance ranges. As evident from Fig. 4(a), choosing the correct cutoff is essential for optimal performance; choosing an inappropriate cutoff can result in almost a 10× performance penalty.

Similar to Secs. IV B and IV C, the accuracy is only slightly affected by the direct space cutoff distance [Fig. 4(b)]. While the RMSE does slightly increase for smaller cutoff distances, the increase in error stays on the order of 10^−8^. Analyzing the histograms of the per-atom error in energies and forces (Figs. S5 and S6) shows that the errors are again well clustered around 0. The deviations in the error distributions stem from the fact that the spherical t-design grids are only symmetric about the upper and lower hemispheres^15^ and can produce slight differences for different components of the force or the energy depending on the orientation of the long-range cell relative to the atom under consideration. Following the same pattern as Secs. IV B and IV C of the energy error being larger than the force error, we analyzed the normalized errors and found similar results to the previous sections, that the normalized energy and force errors are comparable (Tables S5 and S6).

### E. Consideration of precision

Our implementation of GLST uses double-precision variables for atom positions, cubature nodes and weights, structure factors, and forces and energies. However, as an optimization for speed, we use single-precision trigonometric functions as this provides a speedup of 14% for our benchmark system containing 197 159 atoms (cf. Table I). The single-precision trigonometric functions can lead to additional roundoff error, as evident by the asymptotic behavior of the error for increasing target accuracy threshold [Fig. 3(b)] and by the broader peaks in the single-precision error histograms when compared with the double-precision histograms (Figs. S1–S6). This roundoff error can lead to the system having small net forces, which can be removed using Gram–Schmidt orthogonalization. If GLST were to be used for MD simulations, these small net forces could be removed each step before integration.

## V. CONCLUSIONS

We have developed a parallel CUDA implementation of the GLST algorithm for accelerated electrostatic interactions in MD simulations. We tested the algorithm on multiple salt water boxes of varying dimensions and examined the dependence of its performance and accuracy on the target error threshold and the direct space cutoff. Linear scaling of the performance vs the system size and defined control of the RMSE have been demonstrated. The present implementation does not include the periodic boundary condition, and further improvements are also needed. We are currently developing a force-based GLST algorithm that further reduces the number of cubature nodes.^14^ Additional studies are needed, including optimization, periodic boundary condition, incorporation into a simulation engine, and utilization of multiple GPUs. Nevertheless, given its highly parallelizable nature and fine control of accuracy, we believe that GLST can be a competitive algorithm to adopt especially for simulations of large systems running on computing clusters where large numbers of GPU threads or CPU cores can be utilized.

## SUPPLEMENTARY MATERIAL

The supplementary material includes the histograms of the error and tables of the normalized error described in Sec. IV.

## Data Availability

The CUDA/C++ implementation of the GLST algorithm is available as a standalone library under the BSD 3-Clause License.^16^

## References

[1] W. Hwang, S. L. Austin, A. Blondel, E. D. Boittier, S. Boresch, M. Buck, J. Buckner, A. Caflisch, H.-T. Chang, X. Cheng, Y. K. Choi, J.-W. Chu, M. F. Crowley, Q. Cui, A. Damjanovic, Y. Deng, M. Devereux, X. Ding, M. F. Feig, J. Gao, D. R. Glowacki, J. E. Gonzales II, M. B. Hamaneh, E. D. Harder, R. L. Hayes, J. Huang, Y. Huang, P. S. Hudson, W. Im, S. M. Islam, W. Jiang, M. R. Jones, S. Käser, F. L. Kearns, N. R. Kern, J. B. Klauda, T. Lazaridis, J. Lee, J. A. Lemkul, X. Liu, Y. Luo, A. D. MacKerell, Jr., D. T. Major, M. Meuwly, K. Nam, L. Nilsson, V. Ovchinnikov, E. Paci, S. Park, R. W. Pastor, A. R. Pittman, C. B. Post, S. Prasad, J. Pu, Y. Qi, T. Rathinavelan, D. R. Roe, B. Roux, C. N. Rowley, J. Shen, A. C. Simmonett, A. J. Sodt, K. Töpfer, M. Upadhyay, A. van der Vaart, L. I. Vazquez-Salazar, R. M. Venable, L. C. Warrensford, H. L. Woodcock, Y. Wu, C. L. Brooks III, B. R. Brooks, and M. Karplus, “CHARMM at 45: Enhancements in accessibility, functionality, and speed,” J. Phys. Chem. B 128, 9976–10042 (2024). 10.1021/acs.jpcb.4c04100 39303207 PMC11492285

[2] L. Greengard and V. Rokhlin, “A fast algorithm for particle simulations,” J. Comput. Phys. 73, 325–348 (1987). 10.1016/0021-9991(87)90140-9

[3] B. Kohnke, C. Kutzner, and H. Grubmüller, “A GPU-accelerated fast multipole method for GROMACS: Performance and accuracy,” J. Chem. Theory Comput. 16, 6938–6949 (2020). 10.1021/acs.jctc.0c00744 33084336 PMC7660746

[4] B. Kohnke, C. Kutzner, and H. Grubmüller, “A CUDA fast multipole method with highly efficient M2L far field evaluation,” Biophys. J. 120, 176a (2021). 10.1016/j.bpj.2020.11.1234

[5] P. P. Ewald, “Die Berechnung optischer und elektrostatischer Gitterpotentiale,” Ann. Phys. 369, 253–287 (1921). 10.1002/andp.19213690304

[6] J. W. Perram, H. G. Petersen, and S. W. De Leeuw, “An algorithm for the simulation of condensed matter which grows as the 3/2 power of the number of particles,” Mol. Phys. 65, 875–893 (1988). 10.1080/00268978800101471

[7] T. Darden, D. York, and L. Pedersen, “Particle mesh Ewald: An *N*⋅log(*N*) method for Ewald sums in large systems,” J. Chem. Phys. 98, 10089–10092 (1993). 10.1063/1.464397

[8] U. Essmann, L. Perera, M. L. Berkowitz, T. Darden, H. Lee, and L. G. Pedersen, “A smooth particle mesh Ewald method,” J. Chem. Phys. 103, 8577–8593 (1995). 10.1063/1.470117

[9] A. Y. Toukmaji and J. A. Board, Jr., “Ewald summation techniques in perspective: A survey,” Comput. Phys. Commun. 95, 73–92 (1996). 10.1016/0010-4655(96)00016-1

[10] J. Jung, C. Kobayashi, T. Imamura, and Y. Sugita, “Parallel implementation of 3D FFT with volumetric decomposition schemes for efficient molecular dynamics simulations,” Comput. Phys. Commun. 200, 57–65 (2016). 10.1016/j.cpc.2015.10.024

[11] J. Jung, W. Nishima, M. Daniels, G. Bascom, C. Kobayashi, A. Adedoyin, M. Wall, A. Lappala, D. Phillips, W. Fischer, C.-S. Tung, T. Schlick, Y. Sugita, and K. Y. Sanbonmatsu, “Scaling molecular dynamics beyond 100 000 processor cores for large-scale biophysical simulations,” J. Comput. Chem. 40, 1919–1930 (2019). 10.1002/jcc.25840 30994934 PMC7153361

[12] A. C. Simmonett and B. R. Brooks, “A compression strategy for particle mesh Ewald theory,” J. Chem. Phys. 154, 054112 (2021). 10.1063/5.0040966 33557541 PMC7986272

[13] A. Simmonett, B. Brooks, and T. Darden, “Efficient and scalable electrostatics via spherical grids and treecode summation,” J. Chem. Phys. 162, 224101 (2025). 10.1063/5.0264934 40493039 PMC12158466

[14] W. Hwang, J. E. Gonzales II, and B. R. Brooks, “Gauss-Legendre-spherical-t (GLST) cubature-based factorization of long-range electrostatics in simulations,” J. Chem. Phys. 162, 224102 (2025). 10.1063/5.0264936 40488376 PMC12151553

[15] R. S. Womersley, “Efficient spherical designs with good geometric properties,” in Contemporary Computational Mathematics—A Celebration of the 80th Birthday of Ian Sloan (Springer, Cham, 2018), pp. 1243–1285.

[16] See https://github.com/jeg7/Gauss-Legendre-Spherical-t for the source code for the method described in this work.

